# Non-conventional apoptotic response to ionising radiation mediated by N-methyl D-aspartate receptors in immature neuronal cells

**DOI:** 10.3892/ijmm.2013.1245

**Published:** 2013-01-15

**Authors:** NADA SAMARI, LOUIS DE SAINT-GEORGES, GIUSEPPE PANI, SARAH BAATOUT, LUC LEYNS, MOHAMMED ABDERRAFI BENOTMANE

**Affiliations:** 1 Radiobiology Unit, Molecular and Cellular Biology Expert Group, Institute for Environment, Health and Safety, Belgian Nuclear Research Centre, SCK•CEN, B-2400 Mol;; 2 Laboratory for Cell Genetics, Vrije Universiteit Brussel, B-1050 Brussels, Belgium

**Keywords:** N-methyl D-aspartate receptor, fetus, ionising radiation, calpain, cell death, DNA damage

## Abstract

During cortical development, N-methyl D-aspartate (NMDA) receptors are highly involved in neuronal maturation and synapse establishment. Their implication in the phenomenon of excitotoxicity has been extensively described in several neurodegenerative diseases due to the permissive entry of Ca^2+^ ions and massive accumulation in the intracellular compartment, which is highly toxic to cells. Ionising radiation is also a source of stress to the cells, particularly immature neurons. Their capacity to induce cell death has been described for various cell types either by directly damaging the DNA or indirectly through the generation of reactive oxygen species responsible for the activation of a battery of stress response effectors leading in certain cases, to cell death. In this study, in order to determine whether a link exists between NMDA receptors-mediated excitotoxicity and radiation-induced cell death, we evaluated radiation-induced cell death *in vitro* and *in vivo* in maturing neurons during the fetal period. Cell death induction was assessed by TUNEL, caspase-3 activity and DNA ladder assays, with or without the administration of dizocilpine (MK-801), a non-competitive NMDA receptor antagonist which blocks neuronal Ca^2+^ influx. To further investigate the possible involvement of Ca^2+^-dependent enzyme activation, known to occur at high Ca^2+^ concentrations, we examined the protective effect of a calpain inhibitor on cell death induced by radiation. Doses ranging from 0.2 to 0.6 Gy of X-rays elicited a clear apoptotic response that was prevented by the injection of dizocilpine (MK-801) or calpain inhibitor. These data demonstrate the involvement of NMDA receptors in radiation-induced neuronal death by the activation of downstream effectors, including calpain-related pathways. An increased apoptotic process elicited by radiation, occurring independently of the normal developmental scheme, may eliminate post-mitotic but immature neuronal cells and deeply impair the establishment of the neuronal network, which in the case of cortical development is critical for cognitive capacities.

## Introduction

The adult brain has been considered insensitive to radiation due to the relatively radio-resistant nature of mature neurons which in certain model systems show no signs of injury following exposure up to 22 Gy of X-rays (1). Brain damage induced by prenatal irradiation is however a major concern and an important issue in radioprotection (2–4). One of the most important factors apart from dosage, in determining the nature of the damage to the embryo from exposure to ionising radiation, is the developmental stage. Indeed, the phenomenon of radiosensitivity is usually recognized as the vulnerability of mitotic cells to ionising radiation. However, brain development is characterized by the succession of various critical periods, whose disturbance may have severe consequences. Maturation of post-mitotic neurons and early synaptogenesis are thus one of these critical periods. The proper establishment and functioning of synapses is necessary for normal brain development, thus improper synaptogenesis may disturb the cognitive functions and lead to mental retardation and autism (5–7). Prenatal exposure to toxic agents, including radiation may prevent normal synaptogenesis caused by cell loss (8), which may disturb the cognitive functions. Active early synaptogenesis around week 18 of human gestation (9) and day 16 (E16) in embryonic rats (10) is thus a crucial period during which neuronal cells may be highly sensitive. During brain development, cell death is a natural phenomenon that occurs in order to eliminate cells that did not succeed in establishing strong contacts within the neuronal network (11); however, an abnormal rate of cell death during this period that dramatically reduces the number of neuronal cells within the newly established network may lead to neuroanatomical malformation and cognitive disabilities (12).

High doses of ionising radiation clearly damage immature neuronal cells (13), but also more resistant cells such as neuroblastoma in radiotherapy. At low doses, radiation-induced neuronal death has also been observed through the activation of the P53 signaling pathway, the guardian of the genome and upstream of the classical apoptotic pathway (14). Nevertheless, the mechanisms involved in P53-mediated apoptosis may be more complex and may involve other factors, such as the glutamate N-methyl D-aspartate receptor (NMDAr). Indeed, a correlation between P53 induction and NMDAr activation and their involvement in apoptosis has been proven (15).

NMDAr is highly expressed during brain development and is involved in critical biological processes in brain development such as neuronal modulation and synapse maturation (16). The excessive activation of NMDAr is known to be involved in excitotoxicity, a phenomenon described in various neurode-generative pathologies such as Alzheimer’s and Parkinson’s disease (17,18). The main element of glutamate excitotoxicity is the downstream events of NMDAr over-activation that are mainly related to the altered Ca^2+^ homeostasis and its consequences, including neuronal death (19). Calpain, a Ca^2+^-dependent protease is thus activated downstream and plays a central role in the initiation of the cell death pathway (20).

The block of glutamatergic neurotransmission via the use of dizocilpine (MK-801), a non-competitive NMDAr antagonist has been shown to confer significant protection against brain damage caused by ionising radiation when administered subsequent to exposure to 2.5 Gy of γ-rays and has been shown to confer a dose-dependent protection in the dentate gyrus (21). Based on the above, we investigated, *in vivo* and *in vitro* the radiation-induced apoptosis in fetal cortex. We evaluated the possible role of NMDAr and of the intracellular Ca^2+^ concentration in this process, by using MK-801 which blocks Ca^2+^ neuronal influx, and nimodipine, an L-type Ca^2+^ channel blocker.

Furthermore, in order to investigate the possible involvement of apoptotic enzyme activation, known to occur at high Ca^2+^ concentrations, we examined the protective effect of an inhibitor of calpain on irradiated fetal brains and neurons in cultures. Understanding the cellular and molecular mechanisms may aid in the development of strategies to either increase the radiation tolerance or treat central nervous system (CNS) alterations induced by irradiation.

## Materials and methods

### 

#### Animals

BALB/c mice purchased from Janvier Laboratories (Le Genest-St.-Isle, France) and Wistar R/Cnb rats obtained from Vito (Mol, Belgium) were maintained for breeding in a conventional animal facility under the recognition number LA 1100122 according to the national legislation and the guidance of the Ethics Committee of the Belgian Nuclear Research Centre (SCK-CEN) and the Flemish Institute for Technological Research (Vito) for the care and use of laboratory animals.

The Wistar R/Cnb rats were used for *in vivo* study. The animals were mated between 06:00 and 08:30, and the day of fertilization is referred as day 0 (E0). This short mating duration, 150 min, was used in order to obtain very homogeneous groups of embryos at a similar developmental stage. Mice were used at day 17 (E17) of pregnancy and rats at day 15 (E15).

#### Neuronal culture

Primary cortical neuronal cultures were prepared from BALB/cJ Rj (Janvier Laboratories) mouse fetuses, on embryonic day 17 (E17). Pregnant females were sacrificed by cervical dislocation and fetuses were extracted, mice were decapitated and the heads were quickly placed into a dissection medium of cold Hank’s buffered salt solution containing 0.5% glucose and 2.5 U/ml penicillin/streptomycin (all from Invitrogen, Paisley, UK). The brain cortices from each litter were dissected, pooled (between 6 and 8) and enzymatically dissociated for 20 min at 37°C in dissection medium containing 0.1% Trypsin (Invitrogen) and 10 mg/ml DNase I (Sigma-Aldrich, St. Louis, MO, USA). The reaction was terminated by replacing the enzyme solution with dissection medium containing 10% fetal bovine serum (Invitrogen). Mechanical dissociation was carried out in dissection medium containing 5 mg/ml DNase I by trituration through the pipette tip. Dissociated cells were pelleted by centrifugation at 1500 x g for 5 min at room temperature. Cells were then re-suspended in the plating medium containing minimum essential medium, 1 mM sodium pyruvate, 0.6% glucose, 10% fetal bovine serum and 5 U/ml penicillin/streptomycin (all from Invitrogen). The cells were plated at a density of 1.5×10^5^ cells/well onto 13 mm diameter glass coverslips for microscopy or 3×10^6^ cells per flask of 25 cm^2^ seeding surface for cell lysis preparation. Coverslips and flasks were pre-coated with 100 mg/ml poly-D-lysine (Sigma-Aldrich) in 0.1 M borate buffer pH 8.5 and were incubated for 60 min at 37°C in a humidified incubator containing 5% CO_2_. The medium was then replaced with the serum-free growth medium, consisting of neurobasal medium with 2% B27 supplement, 2.5 mM glutamine, 5 U/ml of penicillin/streptomycin (all from Invitrogen) and 25 nM glutamate (Sigma-Aldrich). Cells were grown for 7 days prior to treatment and irradiation. Half of the growth medium was replaced after 3 days with the same medium without glutamate.

#### Irradiation

Animals and cell cultures were irradiated at room temperature with 250 kV-15 mA, 1 mm Cu-filtered X-rays (Pentak HF420 RX machine), delivered at 5 mGy/sec. The farmer 2570-EMI dosimeter was under the control of the Intercomparison Committee for Dosimetry (former EULEP).

Cells were exposed to low (0.1 and 0.2 Gy) and moderate (0.5 Gy) doses of X-rays. Sham-exposed cells were subjected to the same conditions as the irradiated ones and were considered as the controls. Animals used for *in vivo* study (2–3 pregnant rats/group) were whole-body-irradiated with 0.6 Gy of X-rays.

### Treatments

#### Animal treatment

Rats were divided into 4 groups. At 20 min following irradiation, one of those groups was injected intraperitoneally with a 10 mg/ml saline solution to a dose of 3 mg/kg body weight of dizocilpine (MK-801; Sigma-Aldrich), an NMDAr antagonist. The second group was injected with 32 mg/kg of PD 150606, a calpain inhibitor (Calbiochem, Darmstadt, Germany). The third group was injected with 10 mg/kg body weight of nimodipine (Sigma-Aldrich), an L-type Ca^2+^ channel blocker. The fourth irradiated group was injected with the vehicle (saline solution) and used as the positive control for DNA laddering.

Sham-exposed animals untreated or injected with the vehicle, MK-801, calpain inhibitor or nimodipine were used as the negative controls. An average of 9 embryos was collected per female. The brain cortices of the embryos were examined individually by DNA ladder electrophoresis 3 h following injection.

#### Cell culture treatment

For each treatment, 3 replicates from 3 different mouse litters were used. Cells were treated 2 h prior to irradiation as follows: one group of cell cultures was treated with 10 μM of dizocilpine (MK-801; Sigma-Aldrich), the second group was treated with 30 μM of calpeptin, a calpain inhibitor (Calbiochem), the third group was not treated and used as the positive control of the irradiation effect and the fourth group was sham-exposed and used as the negative control.

Following irradiation, cell cultures were placed back for 1 h in the incubator. The cells were then washed and fresh medium was added. Cells were placed back into the incubator and grown for 24 h until further manipulation.

#### γ-H2AX detection by immunofluorescence microscopy

Irradiated and non-irradiated neurons plated on coverslips were fixed with 4% paraformaldehyde 20 min after irradiation. They were permeabilized using 0.1% Triton X-100 (Sigma-Aldrich) then blocked for 30 min with 3% bovine serum albumin (Sigma-Aldrich) and incubated overnight at 4°C with a primary mouse monoclonal antibody against the phosphorylated form of the histone, H2AX (γ-H2AX) (Abcam, Cambridge, UK) diluted 1:300, followed by incubation with a FITC-linked secondary polyclonal goat anti-mouse antibody diluted 1:300 for 1 h at room temperature. The nuclei were counterstained by incubating the coverslips for 5 min with 0.5 μg/ml Hoechst. Coverslips were mounted on glass slides using the vectashield mounting medium (Vector Laboratories, Peterborough, UK). The images were captured using Nikon Eclipse Ti (an automated inverted wide field epifluorescence microscope) equipped with a ×40 oil immersion objective and a Nikon DS-Qi1Mc camera. Images were taken as 16 different frames/coverslip, 19 plains of depth of 0.6 µm thickness. Images were then analyzed using ImageJA freeware version 1.45 b and the number of nuclei and γ-H2AX spots were counted as previously described (22) using an algorithm (provided by Dr Winnok Devos, Ghent University, Ghent, Belgium). In total, approximately 1,200 nuclei from 3 different coverslips were scored and the number of foci/nucleus was reported. The algorithm also determined the average spot occupancy, the area of the nucleus occupied by one focus. The mean number of foci/nucleus and spot occupancy was calculated for each irradiation dose using GraphPad Prism version 5.00 for Windows (GraphPad Software, San Diego, CA, USA).

#### DNA ladder electrophoresis

DNA was extracted from the cortices of the embryos. Mice were sacrificed at 3 h and 20 min after irradiation, and fetal and mother brains were collected. The cortex areas were dissected and genomic DNA was extracted using the Wizard Genomic DNA kit (Promega, Madison, USA). DNA concentration was determined by spectrophotometry, by measuring the absorbance at 260 and 280 nm. DNA (2 μg) was loaded on a 1% agarose gel. The DNA Molecular Weight Marker XIII (Roche Applied Science, Vilvoorde, Belgium) was used as a reference.

#### Cell death analysis

Treated and non-treated neuronal cultures mounted on coverslips were fixed with 4% paraformaldehyde. The cells were then permeabilized with 0.1% Triton X-100 (Sigma-Aldrich) in 0.1% sodium citrate. Apoptotic cells were identified by TUNEL staining using an *In Situ* Cell Death Detection kit (Fluorescein; Roche Applied Science) according to the manufacturer’s instructions. In brief, cells were incubated with the TUNEL reaction mixture containing terminal deoxynucleotidyl transferase (TdT) enzyme and fluoresceindUTP for 1 h at 37°C in a humidified chamber, followed by washing with PBS 3 times. Nuclei were counterstained with Hoechst 0.5 μg/ml for 5 min for total nuclei number counting. Coverslips were mounted on glass slides using the vectashield mounting medium (Vector Laboratories). TUNEL-positive nuclei were counted in 4 randomly selected large fields in each coverslip. One field consisted of a mosaic of 3×3 stitched images acquired by fluorescence microscopy using Nikon Eclipse Ti (an automated inverted wide field epifluorescence microscope) equipped with a ×20 plan dry objective and Nikon DS-Qi1Mc camera). Images were processed using NIS-element Nikon software. A minimum of 10,000 cells was counted in each condition. TUNEL-positive nuclei (green fluorescence) and total nuclei (Hoechst-positive, blue fluorescence) were analyzed with ImageJA freeware version 1.45 b, using an algorithm (provided by Dr Winnok Devos) that automatically counts the number of nuclei detected in the two fluorescence channels. TUNEL-positive but Hoechst-negative cells were excluded. Apoptotic index was calculated as the percentage of TUNEL-positive cells (positive cells/total cells ×100%).

#### Caspase-3 activity test

Caspase-3 activity was examined in the different conditions using a colorimetric activity assay kit (Millipore, Darmstadt, Germany) according to the manufacturer’s instructions. In brief, cells were directly lysed in the flasks using the lysis buffer provided in the kit and scraped, and then the cell lysate was centrifuged to keep only the cytosolic extract. Protein concentration was assessed using the Quick Start Bradford Protein Assay kit 3 (Bio-Rad, Hercules, CA, USA) and the same total protein concentration in all the samples was used for further manipulation. The samples were then incubated with a mixture provided in the kit, containing Ac-DEVD-pNA, the substrate of caspase-3. The optical density (coloration) resulting from the cleavage of the substrate and the release of pNA, was detected and quantified with a microtiter plate reader (Multiscan Ascent; Thermo Labsystems) at 405 nm. A standard curve was also generated using a series of diluted pNA with known concentrations. The standard samples were processed in the same plate and treated as the other samples. The concentration in μM of the released pNA was calculated by projecting the optical densities on the standard curve.

#### Statistical analysis

Analyses of γ-H2AX, TUNEL and caspase-3 activity were carried out in 2 independent experiments using 3 biological replicates.

Data from the cultures exposed only to radiation were processed using the analysis of variance (one-way ANOVA), followed by Tukey’s test. Statistical significance was achieved at P<0.05. Data from cultures exposed to 2 treatments (radiation + inhibitor of calpain or radiation + blocker of NMDAr) were processed using the two-way ANOVA method followed by the Bonferroni multicomparison test. Statistical significance was achieved at P<0.05.

## Results

### 

#### Low and moderate doses of ionising radiation induce DNA damage in maturing neuronal cells

**Figure 1:**
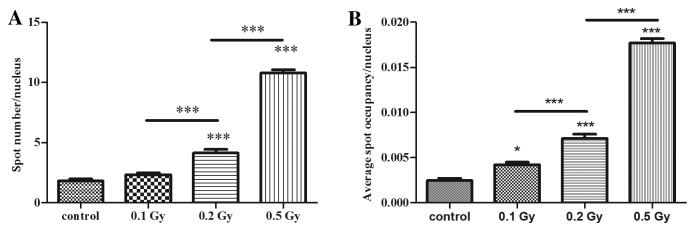
Induction of DNA double-strand breaks in irradiated and non-irradiated 7-day cultured cortical neurons, 20 min following irradiation, revealed by immunofluorescence staining of nuclear γ-H2AX. (A) Number of γ-H2AX foci/nucleus. (B) Quantification of the area of the nucleus occupied by one focus. Data represent the means ± SEM of 2 independent experiments performed in 3 biological replicates. ^*^P<0.05 and ^***^P<0.001 represent significant differences compared to the control or between 2 irradiation doses. One-way ANOVA followed by Tukey’s multicomparison test.

The ability of radiation to induce DNA damage was further assessed with immunofluorescence of γ-H2AX foci assay 20 min following irradiation. Cells treated with 0.2 and 0.5 Gy showed a significantly higher number of γ-H2AX foci than the control cells in a dose-dependent manner. The doses of 0.2 and 0.5 Gy caused 2- and 5-fold more DNA double-strand breaks, respectively than those naturally occurring observed in the control cultures. The dose of 0.1 Gy did not show any significant effect (Fig. 1A).

The spot occupancy that indicates the size of the foci (percentage of nucleus area occupied by one focus) was also calculated in order to overcome the issue of foci clustering that may be counted as only one focus due to spot segmentation issues. This parameter confirmed the result of the first one with a dose-dependent induction of DNA double-strand breaks; however, this parameter showed that 0.1 Gy also induced a significant effect on DNA damage (Fig. 1B).

#### Ionising radiation causes a dose-dependent decrease in cell viability

**Figure 2:**
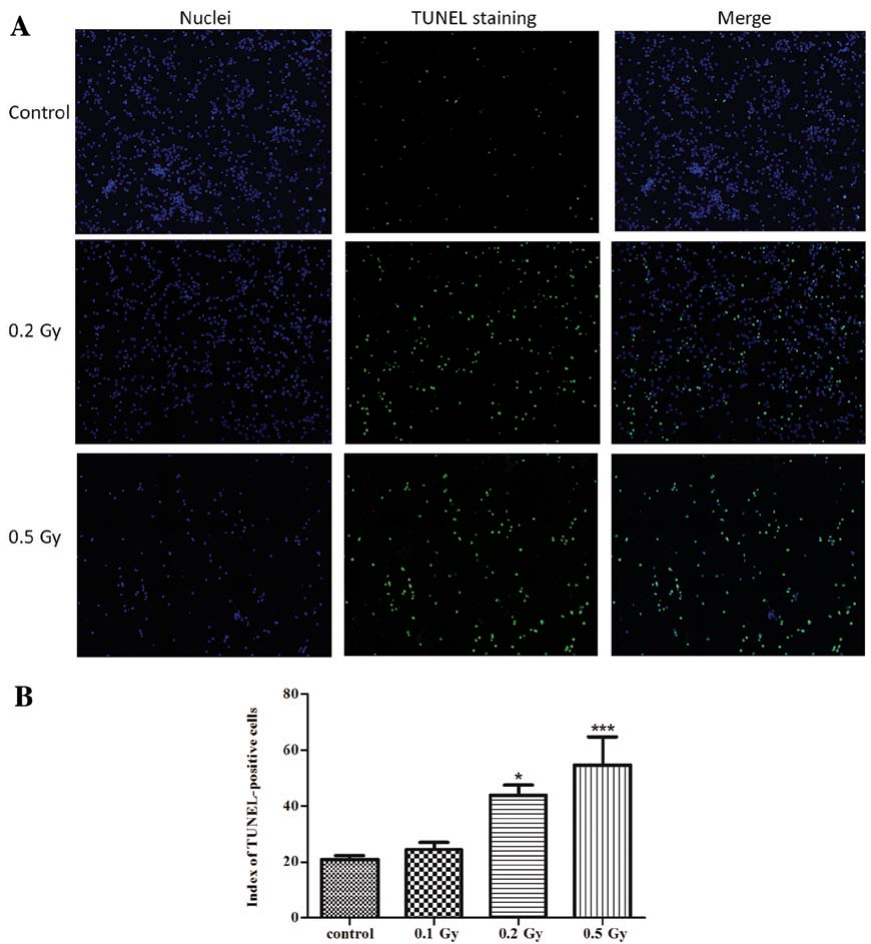
TUNEL-positive cells exposed or not to low and moderate doses of ionising radiation (24 h following irradiation). (A) Fluorescence images of 7-day cultured cortical neurons irradiated and non-irradiated and labeled with TUNEL (green) and Hoechst (blue). (B) Quantification of the number of TUNEL-positive cells in the control and irradiated cultures, plotted as a percentage of TUNEL-positive cells. Labeled cells in each culture were counted in 12 large fields of 3×3 images randomly acquired. Data represent the means ± SEM of 2 independent experiments performed in 3 biological replicates. ^*^P<0.05 and ^***^P<0.001 represent significant differences compared to the control condition. One-way ANOVA followed by Tukey’s multicomparison test.

First we investigated whether low to moderate doses of ionising radiation induce cell death in neurons. DNA fragmentation was then assessed as one of the principal hallmarks of apoptosis using the TUNEL method (Fig. 2). Ionising radiation significantly increased the rate of TUNEL-positive cells after 24 h by 2-fold (P<0.05) compared to the control in the cultures irradiated with 0.2 Gy and by 2.6-fold (P<0.001) in the cultures irradiated with 0.5 Gy, which indicates a dose-dependent induction of apoptosis by ionising radiation. The low dose of 0.1 Gy did not induce any significant increase in apoptosis.

**Figure 3:**
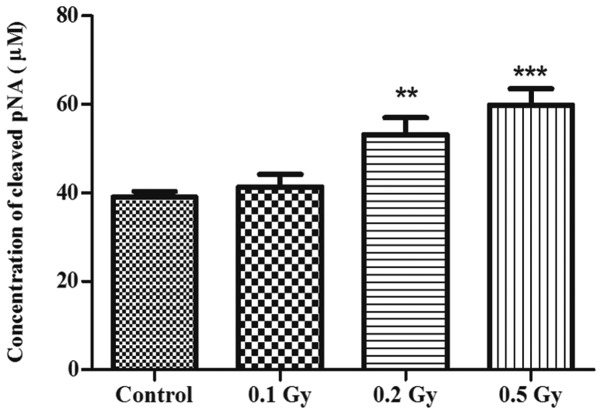
Caspase-3 cleavage activity in cell extracts from irradiated and non-irradiated 7-day cortical neurons, 24 h following irradiation. Caspase-3 activity was measured by determining the ability of cell extracts to cleave the colorimetric substrate, Ac-DEVD-pNA, and plotted as a concentration in μM of the cleaved pNA in the extract. Data represent the means ± SEM of 2 independent experiments performed in 3 biological replicates. ^**^P<0.01 and ^***^P<0.001 represent significant differences compared to the control condition. One-way ANOVA followed by Tukey’s multicomparison test.

In order to corroborate these results and to investigate whether this observed apoptosis was caspase-dependent, the activity of caspase-3, a central factor in apoptosis regulation, was examined by colorimetry, indicating the concentration of the released pNA resulting from the cleavage of Ac-DEVD-pNA by caspase-3 (Fig. 3). The activity of caspase-3 consistently increased by 1.3-fold following exposure to 0.2 Gy (P<0.01) and by 1.5 following exposure to 0.5 Gy (P<0.001) of ionising radiation in comparison with the control, but not following exposure to the lowest dose of 0.1 Gy.

These results clearly indicate the induction of cell death by moderate but not low doses of ionising radiation. Nevertheless, the fold change observed in cell death by TUNEL assay following irradiation was higher than the one observed in caspase-3 activity, suggesting that the radiation-induced cell death assessed in this study was partly caspase-dependent.

#### Radiation-induced apoptosis is mediated by NMDA receptor activation in vivo

Glutamate mediated-excitotoxicity is the most common cause of neuronal death due to a massive entry of calcium into the cell leading to the activation of apoptotic or necrotic pathways.

In an effort to examine whether this mechanism is involved in radiation-induced cell death, the effects of NMDAr were examined *in vivo* by administering a specific NMDAr blocker, MK-801, to a group of pregnant rats and apoptosis induction in E15 fetal cortices before and after treatment was evidenced using the DNA ladder technique.

**Figure 4:**
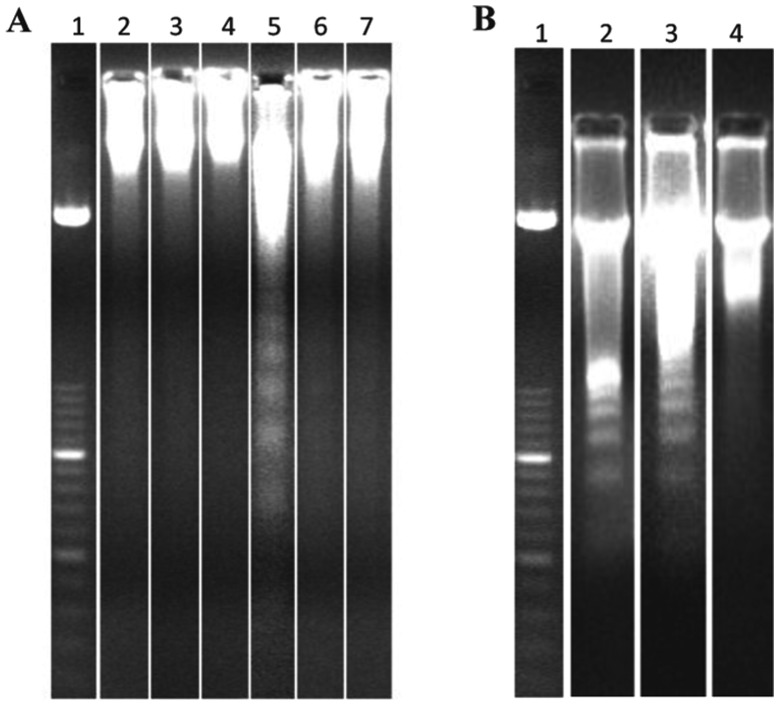
Gel electrophoresis for the detection of DNA fragmentation (DNA ladder) in cortical cells. (A) Animals were treated *in vivo* with 3 mg/kg MK-801 or 32 mg/kg calpain inhibitor 20 min after irradiation with 0.6 Gy of X-rays (lane 1, molecular weight marker; lane 2, sham-exposed; lane 3, MK-801; lane 4, calpain inhibitor; lane 5, 0.6 Gy of X-rays; lane 6, 0.6 Gy of X-rays + MK-801; lane 7, 0.6 Gy of X-rays + calpain inhibitor). (B) Animals were treated *in vivo* with 10 mg/kg of nimodipine 20 min after irradiation with 0.6 Gy of X-rays (lane 1, molecular weight marker; lane 2, 0.6 Gy of X-rays; lane 3, 0.6 Gy of X-rays + nimodipine; lane 4, sham-exposed).

At 3 h and 20 min after exposure, a 0.6 Gy of X-ray dose elicited a clear apoptotic response (Fig. 4A, lane 5 and Fig. 4B, lane 2). This radiation-induced apoptosis could be prevented by injection, 20 min after exposure, of MK-801 (Fig. 4A, lane 6).

The downstream response of NMDAr-mediated cytotoxicity suggests the activation of calpain, a calcium-dependent enzyme. NMDAr was also investigated for its involvement in radiation-induced neuronal death by administering a calpain inhibitor to another group of pregnant rats irradiated with 0.6 Gy of X-rays. Electrophoresis of genomic DNA from cortical cells of the fetuses showed no DNA laddering (Fig. 4A, lane 7) indicating that the calpain inhibitor effectively prevented the fetal cortex from radiation-induced cell death.

The fetal cortices of non-irradiated rats, whether treated or not with MK-801 or calpain inhibitor, did not elicit laddering in DNA electrophoresis (Fig. 4A, lanes 2–4).

In order to dismiss the possible implication of other types of calcium channels in the radiation-induced cytotoxicity, the blockade of L-type Ca^2+^ channels (high threshold Ca^2+^ channels) was performed using the nimodipine blocker. This treatment did not prevent DNA laddering (Fig. 4B, lane 3) indicating that these channels are not involved.

The adult brains (from the mother rats) from all the groups (irradiated, non-irradiated, treated or not with MK-801, nimodipine or calpain inhibitor) did not show any DNA laddering (data not shown). This demonstrates the radiation-resistance of the adult brain. Thus, apoptosis in such adult animals, if any, may exist only at a low level not detectable at least by the method used.

#### Radiation-induced apoptosis is mediated by NMDA receptor activation in vitro

**Figure 5:**
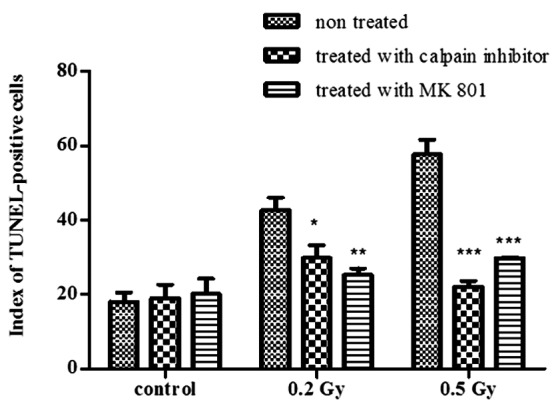
Quantification of the number of TUNEL-positive cells in the control and irradiated 7-day cultures of cortical neurons, treated or non-treated 24 h following irradiation. Cultures were treated 2 h before irradiation with 10 μM of MK-801 or 30 μM of calpain inhibitor. Cell death was assessed using TUNEL and the percentage of TUNEL-positive cells was calculated for the different conditions. Data represent the means ± SEM of 2 independent experiments performed in 3 biological replicates. Two-way ANOVA revealed a significant effect of dose and treatment (P<0.001) and significant interaction between dose and treatment (P<0.001). ^*^P<0.05, ^**^P<0.01 and ^***^P<0.001 represent significant differences compared to the non-treated condition using the Bonferroni post-test.

As regards the high variety of cells that constitute the brain and knowing that NMDA receptors may also be present in glial cells (23), it is very hard to specifically assign the radiation response to one cell type *in vivo*. Thus, *in vitro* study was performed to investigate the specific neuronal role in this response and to avoid any interaction with other cell types. From this perspective the same treatment as for the *in vivo* study was applied to 7-day primary cultures of cortical neurons. Neurons in these cultures were able to establish a network *in vitro* and therefore we chose to use them as a model of neuronal maturation. To assess radiation-induced cell death with or without treatment with MK-801 and calpain inhibitor, TUNEL assay was performed. At 24 h following irradiation, the apoptotic index was significantly reduced using either of the two treatments (Fig. 5) following exposure to 0.2 Gy (P<0.05 for calpain inhibitor and P<0.01 for MK-801) and to 0.5 Gy (P<0.001 for both treatments) and was compared to the control samples (no significant difference), indicating that both treatments prevented radiation-induced cell death.

The non-irradiated cultures were also subjected to the same treatments and showed no effect on the apoptotic index (Fig. 5). These results confirm the *in vivo* findings and assign the response specifically to neurons.

**Figure 6:**
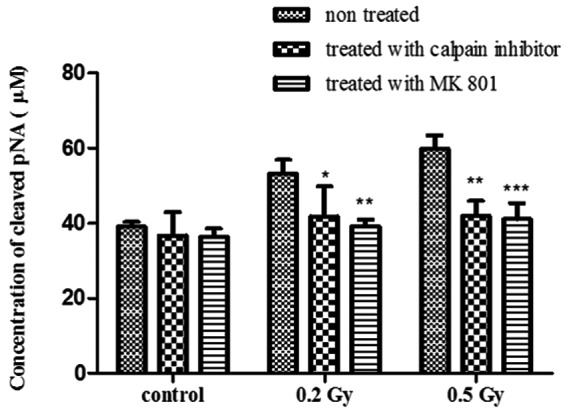
Caspase-3 cleavage activity in cell extracts from control and irradiated 7-day cultures of cortical neurons, treated or non-treated 24 h following irradiation. Cultures were treated 2 h before irradiation with 10 μM of MK-801 or 30 μM of calpain inhibitor. Caspase-3 activity was measured by determining the ability of cell extracts to cleave the colorimetric substrate, Ac-DEVD-pNA, and plotted as concentration in μM of the cleaved pNA in the extract. Data represent the means ± SEM of 2 independent experiments performed in 3 biological replicates. Two-way ANOVA revealed a significant effect of dose (P<0.01) and treatment (P<0.001). No significant interaction between dose and treatment was observed. ^*^P<0.05, ^**^P<0.01 and ^***^P<0.001 significant difference compared to the non-treated condition using the Bonferroni post-test.

In order to verify the downstream pathway used in NMDAr-dependent excitotoxicity following irradiation, caspase-3 activity was also assayed. Consistent with the previous observation using TUNEL assay, caspase-3 activity was also significantly reduced following treatment with MK-801 (P<0.01 with 0.2 Gy and P<0.001 with 0.5 Gy) or with calpain inhibitor (P<0.05 with 0.2 Gy and P<0.01 with 0.5 Gy), compared to the non-treated cultures (Fig. 6). The non-irradiated cultures were also subjected to the same treatments and showed no change in the concentration of cleaved pNA (Fig. 6). This indicates that the response to radiation implicates the activation of caspase-3-dependent pathway.

## Discussion

Cell death as a result of exposure to ionising radiation has been extensively investigated; however, the complexity of the various mechanisms involved in this response remains a key topic of interest. DNA is known to be the most critical radio-sensitive component of cells, directly targeted by radiation or indirectly via water radiolysis that produces reactive oxygen species. These products are responsible for the induction of damage to DNA, including double-strand breaks (24,25), the most damaging lesion that can lead, in the case of repair failure, to cell death, particularly following exposure to low doses of ionising radiation (26). Double-strand breaks were revealed in this study by detecting the phosphorylated histone, H2AX (γ-H2AX), as one of the most effective markers of response to radiation-induced double-strand breaks. This response triggers a signaling cascade by the activation of an important component in double-strand break signaling, the ATM protein kinase. ATM is responsible for the phosphorylation of the H2AX histone and the indirect activation of cell cycle check-points proteins required for cell cycle arrest and DNA repair (27). ATM also regulates the P53 protein, known as the guardian of the genome (28) for its key role in stress response by the induction of cell cycle arrest, DNA repair and apoptosis regulation (29). Its activation has been widely associated with cell death induction (30,31), a phenomenon that was observed in this study following exposure to the moderate doses of 0.2 and 0.5 Gy.

Nevertheless, in the nervous system, multiple pathways leading to neuronal death exist depending on the nature of the stressor, and involve key proteins, such as the Bcl-2 family responsible for the induction of the mitochondrial pathway, leading to the activation of caspase proteins (32) and calpains, calcium-dependent enzymes, involved in cell death induction (33). Evidence of a crosstalk between these pathways makes the process even more complex. A particularity of the neuronal system is the excitability of the cells. The over-activation of NMDA receptors by a high concentration of glutamate, the main excitatory neurotransmitter in the mammalian CNS, causes the cells to die from excitotoxicity, due to a massive entry of calcium ions inside the cell (34). NMDArs are glutamate-gated ion channels, which are selectively activated by the artificial glutamate analog, NMDAr. These channels when open, are highly permeable to Ca^2+^(35).

Attention has been paid to the pathological significance of calcium accumulation in the CNS following insult to the brain, including radiation damage. Excitotoxicity is linked to chronic neurological disorders, including Alzheimer’s and Parkinson’s disease (17,18), and acute CNS insults, including hypoxia/ischemia (36). Over-activation of NMDArs in the brain leads to a sustained influx of Ca^2+^ through NMDA and non-NMDA Ca^2+^ channels. Such disturbances in calcium homeostasis may result in the activation of several calcium-dependent cysteine proteases, including calpain (an intracellular cysteine protease proenzyme activated by autocatalytic cleavage in the presence of high calcium concentrations) and caspases involved in cytotoxicity downstream (37,38). Hence, the selective inhibition of calcium entry by the blockade of ion gated channels to limit neuronal damage after irradiation appears to be an attractive method of evaluating the role of calcium homeostasis in the radiation-induced neurodegenerative processes. We therefore investigated the possible role of NMDAr and Ca^2+^ in the induction of radiation-induced neuronal cell apoptosis.

We showed that a 0.6 Gy of X-ray exposure *in utero*, led to a clear apoptotic response in E15 fetal rat cortices. This apoptotic response was not observed in the different fetal brains of non-irradiated animals used as the controls (Sham-exposed and MK-801, nimodipine or calpain inhibitor-treated animals). The same results were obtained following irradiation of 7-day primary cultures of cortical neurons with 0.2 and 0.5 Gy using the TUNEL test, which indicated radiation-induced cell death. Caspase-3 activity, a key factor in apoptosis induction, was also increased following exposure to the same doses indicating that cell death by apoptosis is caspase-dependent. However, following irradiation, the cell death index was higher than caspase-3 activity, suggesting that other apoptotic mechanisms which are caspase-3-independent may be responsible for this difference in response to radiation. The number of TUNEL-stained cells and caspase-3 activity were not significantly increased in the control cultures (non-irradiated but treated with MK-801 or calpain inhibitor) and the cultures irradiated with the low dose of X-rays.

The apoptotic response including DNA fragmentation (TUNEL) and caspase-3 activation induced in the irradiated cultures with 0.2 and 0.5 Gy was prevented by treatment with MK-801, which selectively blocks NMDAr and neuronal Ca^2+^ influx. This indicates that radiation-induced apoptosis is mediated through NMDAr and is affected by massive entry of Ca^2+^ into the cells.

Calpain was also a good candidate in excitotoxicity-mediated neuronal death; thus, neuronal cultures were treated with calpain inhibitor prior to irradiation. Our results showed that calpain inhibitor prevented the apoptotic response in irradiated cultures, thus supporting our hypothesis of the importance of a calpain-mediated effect in radiation-induced apoptosis in the fetal brain.

Similar results were also observed after *in vivo* treatment of pregnant rats by an injection of MK-801 or calpain inhibitor 20 min following exposure to 0.6 Gy of X-rays. Both treatments prevented DNA laddering, indicating that they can protect the fetal brain from apoptotic response. The *in vivo* experiment also allowed us to eliminate the implication of other Ca^2+^ channels in this radiation-induced excitotoxicity, such as the L-type, high threshold and voltage-dependent Ca^2+^ channels. The blockade of these channels by nimodipine did not prevent irradiation-induced DNA laddering; Therefore, it cannot protect the fetal brain from radiation-induced apoptosis, indicating that the sensitivity of the fetal brain to Ca^2+^ influx through NMDA channels is specific and indicates a particular radiosensitivity of the cell bearing these receptors. Thus, apoptosis induced in immature neurons, by activation of Ca^2+^-dependent proteolytic enzymes such as calpain, plays a key role in the radiation-induced damage of the developing fetal brain.

Our results showing the protective effect of either MK-801 or calpain inhibitor on radiation-induced apoptosis in the fetal cortex and *in vitro*, specifically in established neuronal network of 7-day cultured cortical neurons, further suggest the involvement of various pathways leading to neuronal cell death following exposure to low and moderate doses of ionising radiation.

Indeed, the activation of caspase-3 that was observed following irradiation is a classical response to Ca^2+^ influx, responsible for apoptosis induction by the cleavage of several proteins involved in this process. The inhibition of caspase-3 protects cortical neurons from NMDAr-induced apoptosis (38). The activation of caspase-3 has been reported to be a downstream effector of mitochondrial disruption following the release of cytochrome c (38,39) and is involved in the execution phase of apoptosis.

On the other hand, calpain is involved in several actions following the entry of calcium. Calpain is a proteolytic enzyme directly activated by calcium entry (40) and is mainly known for its capacity to cleave cytoskeletal proteins, such as α-spectrin, a phenomenon that suggests its important role in various neurodegenerative diseases (41). Attention has been paid to the novel roles of calpain in the excitotoxicity phenomenon. It has been found to contribute to the further disturbance of calcium homeostasis by cleaving different substrates involved in calcium extruding, such as the Na^+^/Ca^2+^ exchanger and sarcoplasmic/endoplasmic reticulum calcium ATPase (42,43) or in cytosolic calcium homeostasis, such as the protein phosphatase calcineurin (44). When activated following the cleavage by calpain, the latter triggers downstream effectors known to induce apoptosis, including cytochrome c release from the mitochondria, leading to caspase-3 activation. This has been further proven by the overexpression of 48-kDa calcineurin A (truncated active form), that has been shown to induce an increase in caspase-3 activity and TUNEL-positive apoptotic cells (44). The same finding has been reported using a Parkinson’s disease model, where caspase-3 activation was calpain-dependent (45). A recent study also established a link between the calcium-dependent activation of calpain and the induction of apoptosis via caspases-12, 9 and 3 (46). Our results showing a decrease in caspase-3 activity and DNA fragmentation following treatment with calpain inhibitor also confirm these findings, which permit us to establish a link between calpain and caspase-3 activity, a link that has not always been clear since these two enzymes were believed to be involved in two independent pathways ultimately leading to cell death. Other studies had even described caspase-3 as being directly activated following cleavage by calpain (47,48), indicating another contribution of calpain to the apoptotic induction of caspase-dependent apoptosis.

Our results also demonstrate a radiation-induced DNA damage by detecting double-strand breaks. This damage was shown to proportionally increase with the dose. Such damage is believed to enhance the expression of P53 protein which plays a key role in apoptosis induction (49) through the activation of Bax, a pro-apoptotic protein (50). A P53-dependent activation of Bax has also been shown to be involved in NMDAr-mediated neuronal death (51). Of note, it has been found that calpain activity may be induced following DNA damage and furthermore leads to the activation of P53 (52,53), indicating another role of calpain in the induction of caspase-dependent apoptosis via the activation of P53 response following DNA damage. Furthermore, the fact that the inhibition of calpain in our study almost completely prevented cells from radiation induced-apoptosis, including the fraction of cells that died independently from caspase-3 activation, leads us to suggest an involvement of calpain in both caspase-dependent and -independent pathways.

These studies together with our results indicate a central role of calpain in radiation-induced excitotoxicity, but also indicate an evident crosstalk of several cell death pathways. These interactions and their nature (synergistic or competitive), remain poorly understood; thus investigating these interactions is of high interest for the elaboration of neuroprotective therapies for neurodegenerative diseases caused by excitotoxicity and this study opens new perspectives for radiation protection of the developing brain.

Our results reveal a new non-conventional radiation-induced cell death pathway, involving the excitotoxicity principle mediated by NMDAr activation, not dependent on direct radiation DNA damage. This pathway involves the activation of calpain enzyme but also caspase-3 activation, suggesting the eventual direct or indirect interaction of these two proteins and their respective classical pathways. P53 activation by calpain following radiation-induced DNA damage remains a hypothesis that requires further investigation.
